# Supplementary material for: Collaborative Response of the Host and Symbiotic Lignocellulytic System to Non-Lethal Toxic Stress in Coptotermes formosanus Skiraki
Source: Insects. 2021 May 31;12(6):510. doi: 10.3390/insects12060510 (PMC8227567; doi:10.3390/insects12060510)
Supplement: Supplementary file 1 [file insects-12-00510-s001.zip › Figure S1.pdf]

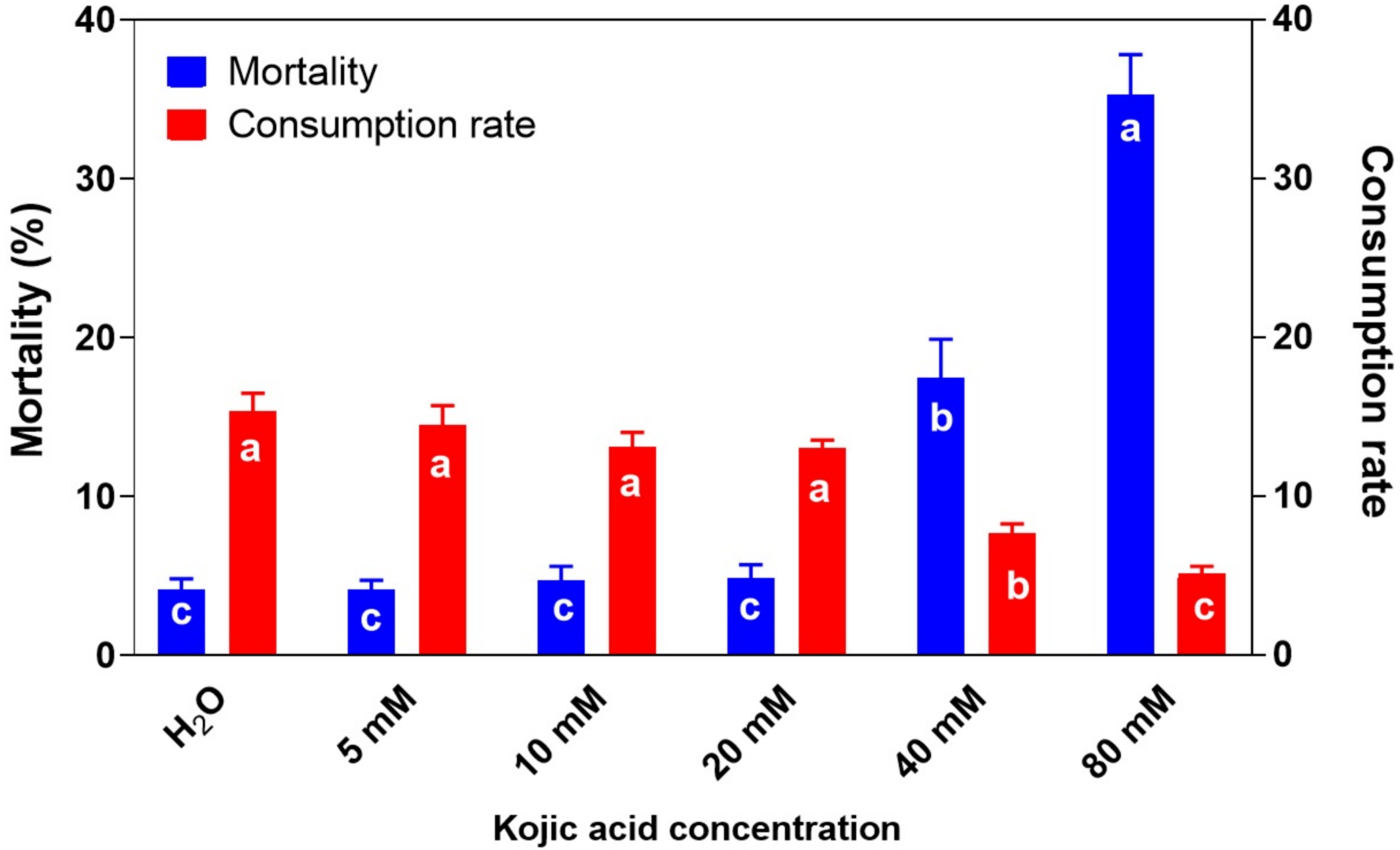

Figure S1: Effects of kojic acid treatment concentration on termite mortality and consumption rate. Data represent the mean ± standard error of the mean (SEM). Significant differences in the mortality and consumption rate between kojic acid treatment and distilled water control were based on statistical analysis using one-way ANOVA method ($p < 0.05$). Consumption rate is defined as mg grams of filter of 80 termites per day.
